# Predictors of polypharmacy among elderly Thais with depressive and anxiety disorders: findings from the DAS study

**DOI:** 10.1186/s12877-018-1001-2

**Published:** 2018-12-12

**Authors:** Nahathai Wongpakaran, Tinakon Wongpakaran, Thanitha Sirirak, Rewadee Jenraumjit, Surin Jiraniramai, Peerasak Lerttrakarnnon

**Affiliations:** 1 Department of Psychiatry, Faculty of Medicine, Chiang Mai University, Chiang Mai, 50200 Thailand; 2 Department of Family Medicine and Preventive Medicine, Faculty of Medicine, Prince of Songkla University, Hat Yai, 90112 Thailand; 3 Department of Pharmaceutical care, Faculty of Pharmacy, Chiang Mai University, Chiang Mai, 50200 Thailand; 4 Department of Family Medicine, Faculty of Medicine, Chiang Mai University, Chiang Mai, 50200 Thailand

## Abstract

**Background:**

Polypharmacy is a geriatric syndrome defined variously as the use of potentially inappropriate drugs and/or the concurrent use of multiple medications including prescription and over-the-counter drugs. An association has been shown between polypharmacy and physical health, increased morbidity and increased mortality. However, there is little information regarding the association between polypharmacy and physical disease, personality trait and mental health problems in elderly. The aim of this study was to investigate potential predictive psychosocial factors related to polypharmacy in elderly Thai people.

**Methods:**

The study analysed the secondary data from the Depressive Disorders, Anxiety Disorders, Suicide Risk and Associated Factors Among Elderly Thai People Program (DAS Study) which was funded by National Research Council of Thailand and conducted between January 2012 and April 2013. Demographic and baseline clinical characteristics including sex, age, education, living alone or with others, access to health care privilege and monthly income were described. The number of medication, physical diseases and mental health problems (i.e. depression, anxiety, and personality trait of neuroticism) were analyzed using descriptive statistics, chi-square and proportional odds logistic regression.

**Results:**

The 803 participants consumed an average of 2.13 prescribed medicines daily (SD 1.46, median = 2). The largest group used 3 medications (18.6%). Predictors found to be associated with polypharmacy in the logistic regression model included hypertension (OR = 1.985, 95% CI = 1.420–2.775), anxiety disorder (OR = 4.402, 95% CI = 2.630–7.367), number of diseases (OR = 2.140, 95% CI = 1.874–2.445), depressive disorder (OR = 1.470, 95% CI = 1.080–2.001), diabetes mellitus (OR = 1.864, 95% CI = 1.122–3.098) and dyslipidemia (OR = 0.511, 95%CI = 0.325–0.803).

**Conclusions:**

The prevalence of polypharmacy among Thai elderly was relatively high compared to other related studies. Several aspects should be taken into consideration before starting an additional medication in elderly patients. In addition to the number of physical disease that leads to polypharmacy, general practitioners should be aware of anxiety, depression, and personality trait of neuroticism that may be related to polypharmacy. Early detection for such condition as well as non-pharmacological intervention could be one way to help reduce polypharmacy in the elderly.

## Background

Polypharmacy is defined as the use of potentially inappropriate drugs and/or as the concurrent use of multiple medications including prescription and over-the-counter drugs [1–5]. The exact number of medications indicating polypharmacy has been defined variously, e.g., 2 or more [1], 4 or more [6], 5 or more [1, 3, 7–11], 6 or more [2, 12–14] and excessive polypharmacy (defined as ≥10 medications) [15] . It has been suggested that based only on the number of medications, five or more items per day could be considered polypharmacy [1]. Polypharmacy has been shown to have an association with falls; including fall risk factors and fall-related injuries, adverse drug events [6, 16, 17], potentially inappropriate medications (PIMS), potential drug-drug interactions (PDDIs), increased healthcare utilization [7], increased hospitalization [6] and increased mortality [14] as well as a variety physical symptoms including decreased cognitive function, poor quality of life and increased risk of dying [6, 7, 10, 18]. Polypharmacy is common among the elderly. The prevalence of polypharmacy in the elderly (≥ 6 drugs) has been reported to be 86.4% in Korea [12], 29.4% in US [2] and, 83.5% in Taiwan [19]. In US, the prevalence of elderly using eight or more drugs was report to be 15.7% [4]. Using a definition of five or more drugs, the prevalence of polypharmacy was 29% among elderly Thais [8] and 26.7% in older adult in Germany [11]. At an internal medicine outpatient clinic of a tertiary care hospital in Thailand, the median number of medicines was 5.6 per patient. Using the Beers criteria for potentially inappropriate medications (PIMs), 19.2% of elderly patients were classified as PIMs while by the Screening Tool of Older Persons’ Potentially Inappropriate Prescriptions. (STOPP) criteria found 31.5% were classified as PIMs [20]. The prevalence of polypharmacy (five or more drugs daily) in elderly inpatients at the facility was 51.9% with an overall average of 4.9 medications. Moreover, the prevalence increased to 67% at discharge with an average of six medications [21]. A study at Thai tertiary care hospital reported a polypharmacy incidence at discharge of 76.3% [22]. A population-based study found the prevalence of using at least 5 medications daily in the elderly aged between 75 and 85 years increased from 30.6 to 35.8% over a period of 7 years (2005–2011) [23]. A study of elderly people with hypertension found that patients were prescribed an average of 3.08 daily medication [24]. Risk factors found to be associated with increased medication use were include increasing age, Indian ethnicity and a higher number of comorbidities specifically those diagnosed with cardiovascular, endocrine and gastrointestinal disorders [7]. Frailty, multimorbidity, obesity, decreased physical and mental health status have been identified as risk factors for excessive polypharmacy in older multimorbid populations in primary care [15]. In a study of Korean elderly [12], predictors for polypharmacy identified were male gender, age 70–84, Medical Aid, and having a larger number of chronic conditions. Study found that polypharmacy was more frequent with females than with males [4]. A study in Germany [11] found factors associated with polypharmacy included breathlessness, hypertension, dependence for basic and instrumental activities of daily living, low subjective health and disagreement between doctors and patients regarding medications. Little has been published regarding the prevalence of polypharmacy in the elderly who present themselves to geriatric mental health clinics. Depression has been shown to be related to polypharmacy. Another study found that depression was a significant independent predictive factor for polypharmacy, occurring nearly 6 times more frequently in people with depression than in individual without depression [25]. Apart from depression, little is known regarding the role of anxiety disorders in polypharmacy. The researchers in the present study were also interested in investigating a common personality trait which is related to depression and anxiety in the elderly, neuroticism [26, 27]. Whether neuroticism is related to polypharmacy has never been reported.

The authors aimed to identify predictive factors of polypharmacy in elderly Thais who were self-referred to tertiary care hospitals. Common physical diseases were examined as well as the presence of mental health problems and neuroticism.

## Methods

### Participants and procedure

This research analysed secondary data from the Depressive Disorders, Anxiety Disorders, Suicide Risk and the Associated Factors among Elderly Thai People Program (DAS Study) which was funded by the National Research Council of Thailand (NRCT) and conducted between January 2012 and April 2013 [28]. The data of DAS Study is stored on the program’s Data Management Unit (DMU) MedRedNet server or DAMUS (Data Archival for Maximum Utilization System, www.damus.in.th/damus), and can be accessed by the program director who granted permission to use the data for this study. All DAS study participants were enrolled who satisfied the study inclusion criteria; age 60 years or more, ability to communicate orally in Thai, volunteered to participate in the study and with at least one of the following symptoms: dysphoric mood, feelings of boredom, sleep problems, eating problems, fatigue, memory problems and unexplained somatic symptoms. Exclusion criteria were any physical illness that significantly affected the interview or completion questionnaires, e.g., dyspnea or disorientation, severe substance addiction, language barriers or other inability to complete the questionnaires and a history of schizophrenia, bipolar disorder or schizoaffective disorders. Study participants were interviewed by research assistant nurses trained to diagnose mental health problems. The nurses were from four different tertiary-care centers across Thailand (Maharaj Nakorn Chiang Mai Hospital, Prince of Songkla University Hospital, Songkla Rajanagarindra Psychiatric Hospital, and Prasart Neurological Institute). The 803 psychiatric elderly patients were evaluated using the Mini-International Neuropsychiatric Interview (M.I.N.I) and the Structured Clinical Interview for DSM-IV TR Diagnosis Axis I disorders (SCID-I/P) to evaluate major Axis I diagnoses according to DSM-IV TR or ICD-10 criteria. Level of neuroticism was assessed using Neuroticism Inventory, a 15-item self-reporting questionnaire with a four-point Likert scale [28].

Demographic data recorded included sex, age, education, living status (alone or with others), access to health care privilege, monthly income and history of diseases. Current physical illnesses, e.g., hypertension, diabetes mellitus, dyslipidemia were categorised according to ICD-10. All participants, whether they met with diagnostic criteria or not, were checked for their medications (current month) on the day of recruitment. This was accomplished by looking at their out-patient records and the medication order systems (either electronic or papers). Participants who were unable to provide the information about their medications were asked to present that information to the investigators later. The number, name, and frequency of medications used daily were recorded. This study was approved by Central Research Ethics Committee of Thailand and the Ethics Committee of each participating hospital.

### Statistical analysis

Demographic and baseline clinical characteristics were analysed. Univariate analysis was used to determine the difference between groups using the chi-square test. Because the distribution of the outcome variable (number of drug used) was skewed, the authors employed proportional odds logistic regression (ordinal regression) to assess the independent effect of the predictors on polypharmacy. The number of drug used was categorized into three groups as the dependent variable. Proportional odds logistic regression is a logistic regression model for ordinal categorical outcome variables, which also works well for skewed continuous outcome variables using ranks of data. Predictors of interest (covariates) included in the multivariable model were age, level of education, marital status, income, access to health care privilege, common medical disease (i.e. hypertension, diabetes mellitus, dyslipidemia), depressive disorder, anxiety disorder, and neuroticism trait. These covariates were opted in based on clinical judgment. Age, hypertension, diabetes, dyslipidemia [11], and number of disease [4] are known risk factors for the polypharmacy. In addition to the previously mentioned socio-demographic variables, access to health care privilege was an important independent variable in the Thai sample. All analyses were conducted using IBM SPSS version 22 (IBM Corp., Armonk, N.Y., USA) and Stata14 (StataCorp., College Station. TX., USA).

## Results

Demographic data are shown in Table 1. Two-thirds of the participants (67.6%) were aged 65 years or older. The majority were females. Physical illnesses and psychological factors such as depressive disorders, anxiety disorders, and neuroticism personality are also reported. Table 2 shows the number of medications used. Thirty-two (4.0%) of participants who reported no medication use in the previous past month. The mean of number of medication was 2.13 (SD =1.46) and the median was 2. The largest group of participants (18.6%) received 3 medication per day, while 5.4% used more than 10 medications (Table 2).

**Table 1:** General Characteristics of Participants from the Depressive Disorders, Anxiety Disorders, Suicide Risk and the Associated Factors Among Elderly Thai people Program (DAS Study)

General characteristics	Frequency (percent)
Sex	
• Female	561 (69.9)
• Male	242 (30.1)
Age (years)	
• 60–64	260 (32.4)
• 65–74	358 (44.6)
• 75 and more	184 (23.0)
Education level	
• Primary school	498 (62.0)
• High school	192 (23.9)
• Bachelor and higher	113 (14.1)
Living status	
• Alone	294 (36.6)
• With others	509 (63.4)
Monthly income (USD; 1 USD = 32.76 Baht)	
• < 152.62	480 (59.8)
• 152.62–305.25	112 (13.9)
• > 305.25	211 (26.3)
Health care privilege	
• Self or private	132 (16.4)
• Civil or state	491 (61.1)
• Universal coverage or social welfare	180 (22.4)
Depression	
• Yes	190 (23.7)
• No	613 (76.3)
Hypertension	
• Yes	205 (25.5)
• No	598 (74.5)
Diabetes mellitus	
• Yes	53 (6.6)
• No	750 (93.4)
Dyslipidemia	
• Yes	61 (7.6)
• No	742 (92.4)
Anxiety disorder	
• Yes	51 (6.4)
• No	752 (93.6)
Neuroticism	
• Low	216 (26.9)
• High	587 (73.1)

**Table 2:** Numbers and percentages of participants by number of medications used and number of diseases

Number of medications/ diseases	Number (%) of participants by number of medications	Number (%) of participants by number of diseases
0	32 (4.0)	0
1	58 (7.2)	349 (45.4)
2	143 (17.8)	201 (26.1)
3	149 (18.6)	104 (13.5)
4	104 (13.0)	50 (6.5)
5	87 (10.8)	34 (4.4)
6	66 (8.2)	22 (2.9)
7	47 (5.9)	5 (0.7)
8	28 (3.5)	2 (0.3)
9	29 (3.6)	1 (0.1)
10	17 (2.1)	0
More than 10	43 (5.4)	1 (0.1)

Participants were divided into 3 groups based on the number of drugs used: including 1–2 drugs (25.0%), 3–5 drugs (42.4%) and 6 drugs or more (23.3%). Anxiety (*p* ≤ 0.001), hypertension (*p* ≤ 0.001), diabetes mellitus (*p* ≤ 0.001), health care privilege (*p* ≤ 0.001), living with others (*p* ≤ 0.05) and depressive disorder (*p* ≤ 0.05) were statistically significant different among the drug groups. Participants who had a higher number of diseases (p ≤ 0.001), had hypertension (*p* ≤ 0.001), had depressive disorder (p ≤ 0.05), had anxiety disorder (*p* ≤ 0.05) and were living with others (p ≤ 0.05) used 3–5 drugs in contrast to 1–2 drugs. This result was statistically significant. Participants who had a higher number of diseases (*p* ≤ 0.001), hypertension (*p* ≤ 0.001), anxiety disorder (*p* ≤ 0.001), depressive disorder (*p* ≤ 0.05), dyslipidemia (*p* ≤ 0.05) and were living with others (*p* ≤ 0.05) were statistically significantly more likely to use more than 5 drugs than 1–2 drugs (Table 3).

**Table 3:** General characteristics of the elderly by medication use

General characteristics and diseases	Percent of drugs used by group			χ^2^ (df)	*P*-value
	1–2 drugs	3–5 drugs	6 drugs or more		
• Depressive disorder	15.5	26.2	28.3	11.50(2)	0.002
• Anxiety disorder	3.9	43.1	52.9	23.05(2)	< 0.001
• Hypertension	4.3	27.6	43.9	96.96(2)	< 0.001
• Diabetes mellitus	1.3	4.1	15.7	44.64(2)	< 0.001
• Dyslipidemia	7.7	9.1	5.2	2.98(2)	0.225
Health care privilege				24.14(4)	< 0.001
• Self or private	21.9	18.3	7.9		
• Civil or state	15.5	25.0	20.7		
• Universal coverage or social welfare	24.9	22.7	19.7		
Education				5.43(4)	0.246
• Less than primary school	77.6	70.2	69.8		
• High school	10.2	14.0	11.5		
• Bachelor and higher	12.2	15.8	18.8		
Monthly income (USD; 1 USD = 32.76 Baht)				9.28(4)	0.054
• < 152.62	56.9	63.0	56.9		
• 152.62–305.25	19.0	11.4	12.4		
• > 305.25	24.1	25.6	30.7		
Age group (years)				3.38(4)	0.497
• 60–64	33.5	31.2	33.2		
• 65–74	44.2	47.6	40.6		
• More than 74	22.3	21.2	26.2		
Living with spouse status				13.11(2)	0.001
• Alone	28.3	36.8	44.5		
• With others	71.7	63.2	55.5		
Number of diseases (Mean ± SD)	1.13 ± .34	1.77 ± .82	3.54 ± 1.72	290.89^a^(2)	< .001
Neuroticism score	40.83 ± 11.37	43.57 ± 12.72	45.90 ± 13.53	8.94(2)	< 0.001

^a^*F* statistic

Predictors associated with polypharmacy were calculated by proportional odds logistic regression. The model exhibited a goodness of fit (χ^2^ = 1187, df = 1279, *p* = .967). Significant predictors included hypertension (OR = 1.985, 95%CI = 1.420–2.775, p ≤ 0.001,), anxiety disorder (OR = 4.402, 95%CI = 2.630–7.367, *p* <  0.001), number of diseases (OR = 2.140, 95%CI = 1.874–2.445, p ≤ 0.001), depressive disorder (OR = 1.470, 95%CI = 1.080–2.001, *p* = .014), diabetes mellitus (OR = 1.864, 95%CI = 1.122–3.098, *p* = 0.016,) and dyslipidemia (OR = 0.511, 95%CI = 0.325–0.803, *p* = 0.014). The model yielded the Cox and Snell pseudo-R square of 0.497 (Table 4). Among the predictors, anxiety disorder showed the highest odds ratio, indicating that participant with anxiety disorder were four times more likely to use more drugs. On the contrary, those who had dyslipidemia tends to use less drugs.

**Table 4:** Predictors of the number of drugs used

Variables	Adjusted	95% Confidence Interval		Adjusted *p* value
	Odds ratio	Lower Bound	Upper Bound	
Age	0.992	0.973	1.012	0.432
Gender	0.932	0.701	1.241	0.631
Education	1.170	0.941	1.454	0.157
Income	0.938	0.779	1.13	0.501
Health care privilege	1.028	0.936	1.129	0.558
Hypertension	1.985	1.420	2.775	< 0.001
Diabetes mellitus	1.864	1.122	3.098	0.016
Dyslipidemia	0.511	0.325	0.803	0.004
Depressive disorder	1.470	1.080	2.001	0.014
Anxiety disorder	4.402	2.630	7.367	< 0.001
Neuroticism	1.127	0.344	1.393	0.410
Number of diseases	2.140	1.874	2.445	< 0.001

## Discussions

Our study investigated the prevalence and predictors associated with polypharmacy in the elderly at primary care clinics and psychiatric clinics of tertiary care hospitals within the DAS program. The incidence using five or more drugs among study participants (39.5%) was higher than that found in a community in Thailand (29%) [8] and in primary care facilities in Germany (26.7%) [11]. The percentage of participants who used six or more drugs 28.6% approximately the same as 29.4% in a US study [2] but less than 86.4% found in the Korean elderly [12] and less than the 83.5% in the Taiwanese elderly [19].

Most common number of medications used was three. Rather than using specific numerical definition of polypharmacy. This study evaluated the actual number of drugs used. The socio-economic factors included in the study differed by region within the country. Living with a spouse was associated with higher drug usage. Consistent with other related studies, number of diseases, hypertension, and diabetes mellitus as well as anxiety and depressive disorders were associated with higher drug used [4, 7, 12]. The exception was dyslipidemia which, instead of being a consistent predictive factor of increased drug use was associated with a lower level of use of (six of more drugs) in the multivariable logistic model. The reason for that is not yet clearly understood. We assume that it could be associated with some factors. One possible explanation is that dyslipidemia is known to be undertreated in older adult [29], and to be related to many clinical conditions, e.g., metabolic syndrome and cardiovascular condition. That knowledge might lead to an awareness and lead to improved drug management by attending physician, especially, in tertiary care facility such as those where this study was conducted. The characteristics of physicians including experience and knowledge of physician in those facilities related to geriatric patients could also potentially lead to a decrease in prescription of drugs for elderly patients with more complicated condition [30]. That, in turn suggest that clinicians’ knowledge is a key to reducing polypharmacy. This is in agreement with previous studies which have reported and association, albeit inconsistent, between dyslipidemia and depression [31, 32].

Anxiety disorder and depressive disorder were found to be associated with higher medication use in this study, with anxiety disorder appearing to be more influential than depressive disorder. The relationship of depression and polypharmacy is supported by previous studies, one even claimed that depression was a better independent predictor of polypharmacy than other comorbid diseases. [25, 33, 34] Demonstration that anxiety disorder has important role in polypharmacy, was one of the early findings. How anxiety and depressive disorders influenced polypharmacy has not yet been clearly explained. Those condition might aggravate existing physical disease and produce more symptoms (e.g., chest pain, joint pain) that lead to receiving more prescription for symptom reduction [35, 36]. Effort should be focused on how to identify these mental health problems and how to alleviate their impact. In addition early detection, non-pharmacological approach could also be used to avoid adding more medications to their treatment.

Higher medication use in this study was not found to be associated with age, sex, or with endocrine or gastrointestinal diseases [4, 7, 12]. One limitation of this study was that Medical Aids status [4] was not recorded in our study.

In terms of the participants’ personality traits, neuroticism was chosen for studied as this kind of personality plays a strong role in predicting late-life depression. Individuals with high level of neuroticism are characterized by having emotional instability, which can include anxiety, depression, being easily distressed, insecurity, impulsiveness, and vulnerability to stress. Moreover, neuroticism might influence the tendency of participants to manifest more somatic symptoms and as a result to receive more medication for their complaints as in a previous study [37]. However, neuroticism was not a predictor in the model in this study due to the mediation effect of anxiety and depression [27]. The best of our knowledge, this is the first study to report on the relationship between neuroticism and polypharmacy.

Strengths of our study include the involvement of multi-centers (in the northern, central and southern part of Thailand), inclusion of a large sample size of elderly, and the use of standardized measurements of representative mental health problem of the elderly in tertiary care hospital. The study shed light on the importance of depression disorder, anxiety disorder, and neuroticism were. However, a limitation was that some potentially important variables were not including in the DAS study (e.g., frailty, quality of life). Additionally, because of the cross-sectional design of DAS, were not able reach conclusions regarding causality.

There are a variety of processes to help integrate the results of this study into clinical practice. A first step would be to establish a multidisciplinary health care team working as a collaborative prescriber of medications in geriatric clinic. As s second step, a specific chronic patient care protocol should be developed in patient with chronic disease associated with psychiatric disorders (e.g., major depressive disorder, anxiety, behavioural and psychological symptoms of dementia) who are treated with antidepressants or antipsychotic drugs, should be developed. That protocol should includes a list the high-risk medication, as well as prescribing guideline, and deprescribing scripts tailored to Thai geriatric patients with multi-morbidities. Finally, inter-professional conference to share a decision making should be conducted regularly especially for patient who require complex care.

Future studies should investigate additional variables including comprehensive geriatric assessment, potentially inappropriate medications (PIMs) by Beers [38] and PIMs by STOPP [39]. Drug-drug interaction should also be studied and monitored. The present study investigated polypharmacy by looking at the record of medication prescriptions. Additionally, it should not be overlooked that self-medication is common problem in elderly patients in low and middle income countries such as Thailand.

## Conclusions

The prevalence of polypharmacy among elderly patients in psychogeriatric and geriatric clinics in Thailand is high. Predictors of polypharmacy include number of diseases, hypertension, diabetes mellitus, anxiety disorders, and depressive disorder. Before initiating additional medication in elderly patients, the indication should be clearly specified, and several aspects should be taken into consideration. In addition, anxiety and depressive disorder should be identified and appropriately treated, including by non-pharmacological intervention. General practitioners at primary care level should be aware of those predictors.

## Abbreviations

DSM-IV TR: Diagnostic and Statistical Manual of Mental disorders, Fourth edition, Text Revision
ICD: International Statistical Classification of Diseases and related Health Problems
PDDIs: Potential drug-drug interactions
PIMS: Potential inappropriate medications
